# *In vitro* nuclear magnetic resonance spectroscopy metabolic biomarkers for the combination of temozolomide with PI3K inhibition in paediatric glioblastoma cells

**DOI:** 10.1371/journal.pone.0180263

**Published:** 2017-07-13

**Authors:** Nada M. S. Al-Saffar, Alice Agliano, Lynley V. Marshall, L. Elizabeth Jackson, Geetha Balarajah, Jasmin Sidhu, Paul A. Clarke, Chris Jones, Paul Workman, Andrew D. J. Pearson, Martin O. Leach

**Affiliations:** 1 Cancer Research UK Cancer Imaging Centre, Division of Radiotherapy and Imaging, The Institute of Cancer Research and The Royal Marsden NHS Foundation Trust, London, United Kingdom; 2 Divisions of Cancer Therapeutics and Molecular Pathology, The Institute of Cancer Research and The Royal Marsden NHS Foundation Trust, London, United Kingdom; 3 Divisions of Clinical Studies and Cancer Therapeutics, The Institute of Cancer Research and The Royal Marsden NHS Foundation Trust, London, United Kingdom; 4 Cancer Research UK Cancer Therapeutics Unit, Division of Cancer Therapeutics, The Institute of Cancer Research, London, United Kingdom; Instituto de Investigacion Sanitaria INCLIVA, SPAIN

## Abstract

Recent experimental data showed that the PI3K pathway contributes to resistance to temozolomide (TMZ) in paediatric glioblastoma and that this effect is reversed by combination treatment of TMZ with a PI3K inhibitor. Our aim is to assess whether this combination results in metabolic changes that are detectable by nuclear magnetic resonance (NMR) spectroscopy, potentially providing metabolic biomarkers for PI3K inhibition and TMZ combination treatment. Using two genetically distinct paediatric glioblastoma cell lines, SF188 and KNS42, *in vitro* ^1^H-NMR analysis following treatment with the dual pan-Class I PI3K/mTOR inhibitor PI-103 resulted in a decrease in lactate and phosphocholine (PC) levels (P<0.02) relative to control. In contrast, treatment with TMZ caused an increase in glycerolphosphocholine (GPC) levels (P≤0.05). Combination of PI-103 with TMZ showed metabolic effects of both agents including a decrease in the levels of lactate and PC (P<0.02) while an increase in GPC (P<0.05). We also report a decrease in the protein expression levels of HK2, LDHA and CHKA providing likely mechanisms for the depletion of lactate and PC, respectively. Our results show that our *in vitro* NMR-detected changes in lactate and choline metabolites may have potential as non-invasive biomarkers for monitoring response to combination of PI3K/mTOR inhibitors with TMZ during clinical trials in children with glioblastoma, subject to further *in vivo* validation.

## Introduction

Glioblastomas (grade IV astrocytomas) are very aggressive tumours and are one of the leading causes of brain tumour-related deaths in children [1]. Although these tumours are morphologically similar to malignant gliomas that arise in adults, increasing evidence indicates that the molecular pathways activated in brain neoplasms in children substantially differ from those in adults [2–6]. Despite these molecular differences, both adult and childhood malignant gliomas are generally treated similarly post-operatively, with the Stupp regimen of concomitant radiotherapy and temozolomide (TMZ) followed by adjuvant TMZ [7]. However, the published paediatric literature suggests that TMZ may be less effective in high-grade astrocytomas in children compared to adults [8–10]. Recent studies have investigated the differential mechanisms of resistance to TMZ in a series of paediatric cell lines [11, 12]. Results from one study [12] showed that, as in adults, in the majority of paediatric glioblastoma cell lines TMZ resistance was linked to a lack of promoter methylation of the gene encoding the repair protein DNA methyltransferase MGMT (O6-methylguanine-DNA-methyl-transferase). However, in glioblastoma cells not expressing MGMT, resistance to TMZ was shown to be associated with a PI3K–mediated HOX/stem cell gene signature, and this resistance was reversed by inhibition of the PI3K signalling pathway using the dual pan-class I PI3K/mTOR inhibitor PI-103 [13–15]. Thus, combination of TMZ with PI3K inhibition may provide one therapeutic strategy for those children who tumours either have innate resistance to, or which acquire resistance to, TMZ treatment.

Magnetic resonance imaging has impacted greatly on the management of brain tumours; however, the use of tumour size (e.g. RECIST criteria) for the evaluation of treatment response to pathway-based targeted therapies may not be adequate (as a change in brain tumour size may take time to be detected). Functional imaging modalities that probe tissue properties are being increasingly investigated for their use in the clinical monitoring of brain tumours. The main techniques include positron emission tomography (PET), diffusion imaging, perfusion imaging and magnetic resonance spectroscopy (MRS) [16–19]. MRS represents a non-invasive and non-ionizing method of characterizing biological samples on the basis of the metabolic (chemical) content [20]. Proton (^1^H)-MRS is most commonly used clinically owing to its high inherent sensitivity, and the brain is the most studied anatomical region, including for the pre-surgical diagnosis of tumour type and grade, monitoring of treatment response, and evaluation of tumour recurrence [21–23]. Common metabolites measured include: N-acetyl aspartate, total choline (tCho), creatine, myoinositol, lactate, taurine, glutamate+glutamine and lipids; for example, a high level of tCho and its ratio to N-acetyl aspartate is a hallmark of most brain tumours and has been used to distinguish tumours from other lesions [24, 25].

Using NMR spectroscopy, we previously reported decreases in choline metabolites and lactate levels in response to different PI3K pathway inhibitors in paediatric glioblastoma cell lines [26]. In contrast, treatment with TMZ increased levels of phosphocholine (PC), glycerophosphocholine (GPC) and tCho. In this study, we have used two genetically distinct paediatric glioblastoma cell lines KNS42 and SF188, to identify whether a combination of the dual pan-Class I PI3K/mTOR inhibitor PI-103 [13–15] with TMZ would result in metabolic changes detectable by NMR spectroscopy, with potential to be used as a non-invasive method of monitoring response in early phase and subsequent clinical trials in children with glioblastoma. We have also assessed mechanisms underlying the detected metabolic changes.

Our results show that in both KNS42 and SF188 paediatric glioblastoma cells, combination treatment with PI-103 and TMZ resulted in a decrease in PC and lactate, and an increase in GPC levels. Furthermore, the decrease in PC levels was associated with a decrease in the protein levels of choline kinase alpha (CHKA), the enzyme responsible for choline phosphorylation to form PC. A decrease in the expression levels of the glycolytic enzymes hexokinase II (HK2) and lactate dehydrogenase alpha (LDHA) was also observed, suggesting reduced glycolytic flux as a mechanism for the depletion of lactate.

Taken together, our findings provide a step towards identifying MRS biomarkers that may potentially be used to monitor response to combination therapy of PI3K pathway inhibition with TMZ during clinical trials in children with glioblastoma.

## Materials and methods

### Cell culture and treatment

The human paediatric glioblastoma (WHO grade IV) KNS42 and SF188 cell lines were obtained and cultured as previously described [27]. Cell viability was routinely >90%, as judged by trypan blue exclusion. Both cell lines routinely tested negative for mycoplasma by PCR.

Cells were treated with the dual pan-Class I PI3K/mTOR inhibitor PI-103 [13–15] (Sigma; 2xGI_50_), TMZ (Sigma; 2xGI_50_), or simultaneously with both PI-103 and TMZ (each at 2xGI_50_). GI_50_ values (concentrations causing 50% inhibition of proliferation of tumour cells) were determined using the MTS assay [28] following continuous exposure to compounds for 3 doubling times. For NMR experiments, cells underwent trypsinisation and trypan blue exclusion assay following continuous incubation equal to a single doubling time (KNS42 = 48 h, SF188 = 24 h) [26]. The effect of treatment on cell number was monitored by counting the number of viable attached cells in a treated flask and comparing that number with the number of attached cells in a control flask. Cell diameter (d) was measured with Vi-CELL Cell Viability Analyser (Beckman Coulter) and cell volume was calculated using the formula [4/3 л (d/2)^3^].

### Flow cytometry

Cell cycle analysis was performed as previously described [26]. Control and treated cells were harvested by trypsinisation, washed in PBS and fixed in 70% ethanol. Fixed cells were washed and resuspended in PBS supplemented with 10 mg/ml RNase A (Sigma) and 40 mg/mL propidium iodide (Sigma). After 30 minutes of incubation at 37°C, cells were analysed using a BD LSRII flow cytometer (BD, San Jose, CA, USA). The cytometry data were analysed using WinMdi and Cylchred software (University of Wales College of Medicine, Cardiff, UK).

### Immunoblotting

Western blotting was performed as previously described [26]. Cells were lysed in lysis buffer (Cell Signaling) supplemented with a complete mini protease inhibitor cocktail (Roche Diagnostics). Protein concentration was determined using a BIO-RAD assay. Total protein extracts (30μg/lane) were separated electrophoretically in 10% SDS-polyacrylamide gel and transferred onto immobilon-P membranes (Millipore). Immunodetection was performed using anti: pAKT (Ser473) rabbit monoclonal antibody (1:2000; Cell Signaling), total AKT rabbit polyclonal antibody (1:2000; Cell Signaling), pRPS6 (Ser240/244) rabbit polyclonal antibody (1:2000; Cell Signaling), total RPS6 rabbit monoclonal antibody (1:2000; Cell Signaling), HK2 rabbit polyclonal antibody (1:2000; Cell Signaling), PARP rabbit polyclonal antibody (1:2000; Cell Signaling), CHKA rabbit polyclonal antibody (1:2000; Sigma), LDHA goat polyclonal antibody (1:5000; Santa Cruz Biotechnology) and GAPDH mouse monoclonal antibody (1:10000; Merck). Blots were revealed with peroxidase-conjugated secondary anti-rabbit (1:2000; GE healthcare), anti-mouse (1:10000, DAKO) or anti-goat (1:10000, Santa Cruz Biotechnology) antibodies followed by ECL chemiluminescence solution (Amersham Biosciences). Western blotting bands were quantified by densitometry using ImageJ software (https://imagej.nih.gov/ij/).

### *In vitro* ^1^H-MRS of cell extracts

To obtain an NMR spectrum, an average of 3x10^7^ cells in logarithmic phase were extracted from cell culture using the dual phase extraction method, as previously described [26, 29]. Briefly, cells were rinsed with ice-cold saline and fixed with 5 ml of ice-cold methanol. Cells were then scraped off the surface of the culture flask and collected into tubes. Ice-cold chloroform (5 ml) was then added to each tube followed by an equal volume of ice-cold deionized water. Following phase separation, the solvent in the upper methanol/water phase was removed by lyophilisation. Prior to acquisition of the NMR spectra, the water-soluble metabolites were resuspended in deuterium oxide (D_2_O). ^1^H-NMR spectra were acquired at 25°C on a 500 MHz Bruker spectrometer (Bruker Biospin) using a 90-degree flip angle, a 1 s relaxation delay, 10 s acquisition time, spectral width of 12 ppm, 64 K data points, and HDO resonance suppression by presaturation. Metabolite contents were determined by integration and normalised relative to the peak integral of an internal reference (TSP 0.15% in D2O) and corrected for the number of cells extracted per sample and average cell volume.

### Statistical analysis

Data are presented as the mean ± SD and n≥3. Statistical significance of differences was determined by unpaired two-tailed Student’s standard *t*-tests with a *P* value of ≤0.05 considered to be statistically significant.

## Results

### Molecular effects of the combination of PI-103 with TMZ in KNS42 paediatric glioblastoma cells

**Fig 1:**
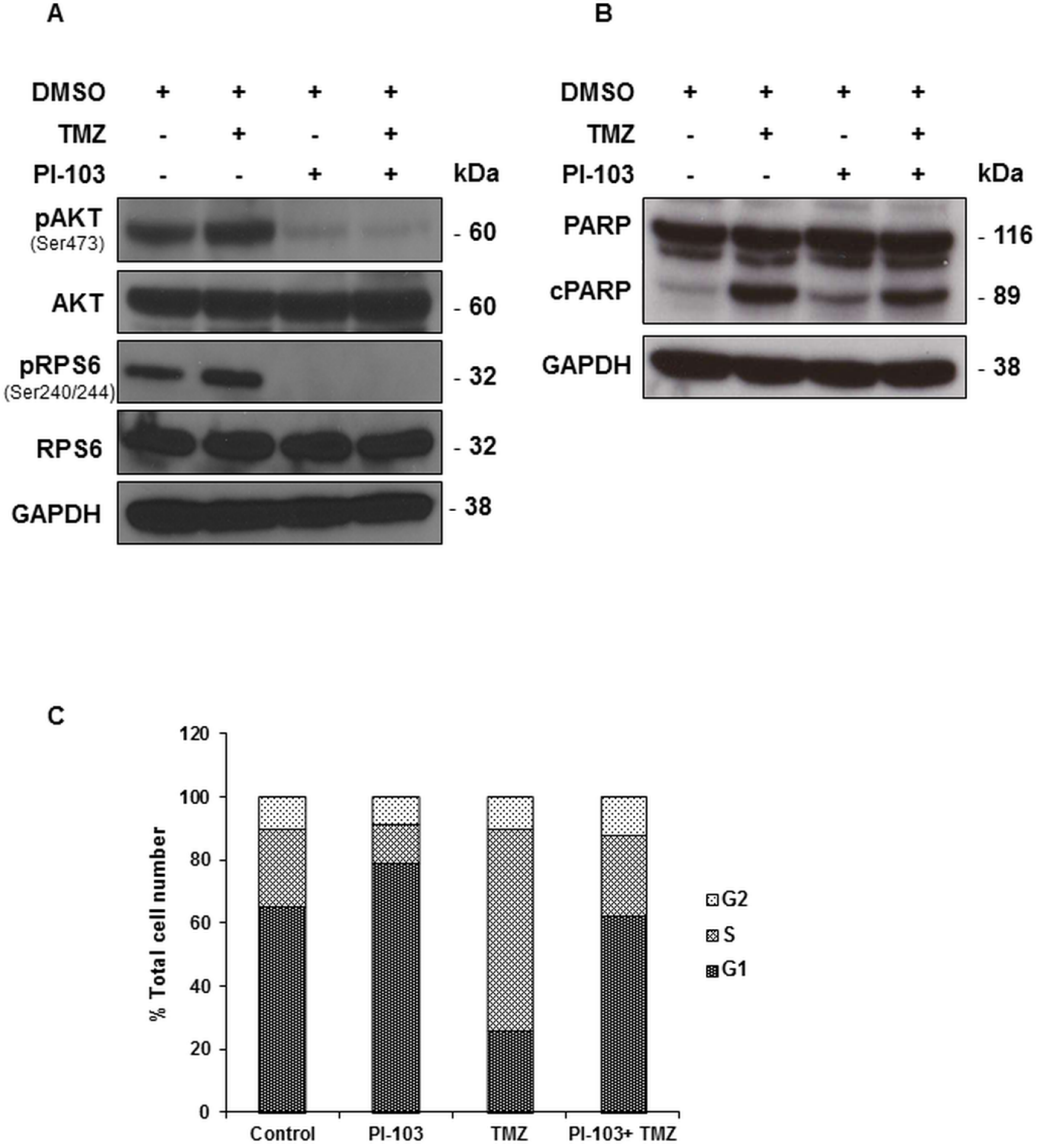
Molecular changes following treatment (48 h) with TMZ, PI-103 or PI-103+TMZ in KNS42 paediatric glioblastoma cells. (A) Representative Western blots showing decreases in molecular markers in the PI3K signalling pathway post-treatment relative to controls. (B) Representative Western blots showing induction of apoptosis as evidenced by the presence of cleaved PARP following treatment with TMZ or the combination of PI-103 with TMZ. GAPDH is used as a loading control. (C) A summary of the cell cycle distribution of control cells (DMSO), or following treatment.

Previous studies have shown that the human paediatric glioblastoma (WHO grade IV) KNS42 cells are wildtype for *PTEN* and *PIK3CA* [12, 27], histone H3.3 (H3F3A) G34V mutant [30] and have no expression of MGMT [11, 12]. In this study, KNS42 cells were treated for 48 h with the dual pan-Class I PI3K/mTOR inhibitor PI-103 [13–15] (2xGI_50_, GI_50_ = 1.4 μM), TMZ (2xGI_50_, GI_50_ = 900 μM), or simultaneously with both PI-103 and TMZ (each at 2xGI_50_). A concentration equivalent to 2xGI_50_ was selected in order to be more representative of clinical conditions and to minimise cytotoxic side effects following the combination of both drugs. Single treatment with PI-103 resulted in inhibition of signalling downstream of PI3K as indicated by decreased phosphorylation of AKT (Ser473) and RPS6 (Ser240/244) in treated cells compared to their controls (Fig 1A, Table 1). Treatment of KNS42 cells with TMZ induced apoptosis as indicated by PARP cleavage detected by immunoblotting with no effects on the PI3K signalling pathway (Fig 1A and 1B, Table 1). Cell cycle analysis (cca) of attached cells following treatment with PI-103 showed an increase in the G1 cell population (79±4% vs. 66±5%, *P* = 0.01) and a decrease in S phase (12±3% vs. 25±2%, *P* = 0.001) compared to control cells, while treatment with TMZ resulted in an increase in the S phase (64±1% vs. 25±2%, *P* = 0.000001) and a decrease in the G1 phase (26±2% vs. 66±5%, *P* = 0.0003; Fig 1C). Immunoblotting analysis showed that PI-103 and TMZ have maintained their different mechanisms of action following combination resulting in the inhibition of signalling downstream of PI3K as indicated by decreased phosphorylation of AKT (Ser473) and RPS6 (Ser240/244) and apoptosis as evident by PARP cleavage (Fig 1A and 1B, Table 1). Cell cycle analysis following combined treatment with PI-103 and TMZ showed that non-apoptotic treated cells had a similar cell cycle distribution to that of control cells (Fig 1C), resulting from the opposing effects of PI-103 and TMZ on the G1 and S phases of the cell cycle. Furthermore, relative to the DMSO-treated controls, the combined agents caused a higher reduction in cell number (down to 54±6%, *P* = 0.001) compared to single agent treatment with PI-103 (down to 79±8%, *P* = 0.02) or TMZ (down to 65±10%, *P* = 0.01). Treatment with PI-103 caused a decrease in the average cell volume (down to 76±6%, *P* = 0.03) relative to control, while no change in cell volume was detected following treatment with TMZ alone or in combination with PI-103 (*P*≥0.2).

**Table 1:** Densitometric analysis of immunoblots from control and treated (48 h) KNS42 paediatric glioblastoma cell extracts.

	pAKT(ser473)/AKT	pRPS6(Ser240/244)/RPS6	cPARP/PARP
**PI-103**	0.1(±0.1)*	0.1(±0.2)*	1.3(±0.6)
**TMZ**	1.0(±0.2)	0.9(±0.3)	5.5(±3.5)*
**PI-103+ TMZ**	0.1(±0.1)*	0.1(±0.1)*	4.6(±1.9)*

Data are expressed as fold changes relative to controls and presented as the mean ± SD, n ≥ 3.

Two-tailed unpaired *t* test was used to compare results in treated cells to controls

*P≤0.05.

### ^1^H-NMR-detected metabolic effects of the combination of PI-103 with TMZ in KNS42 paediatric glioblastoma cells

**Fig 2:**
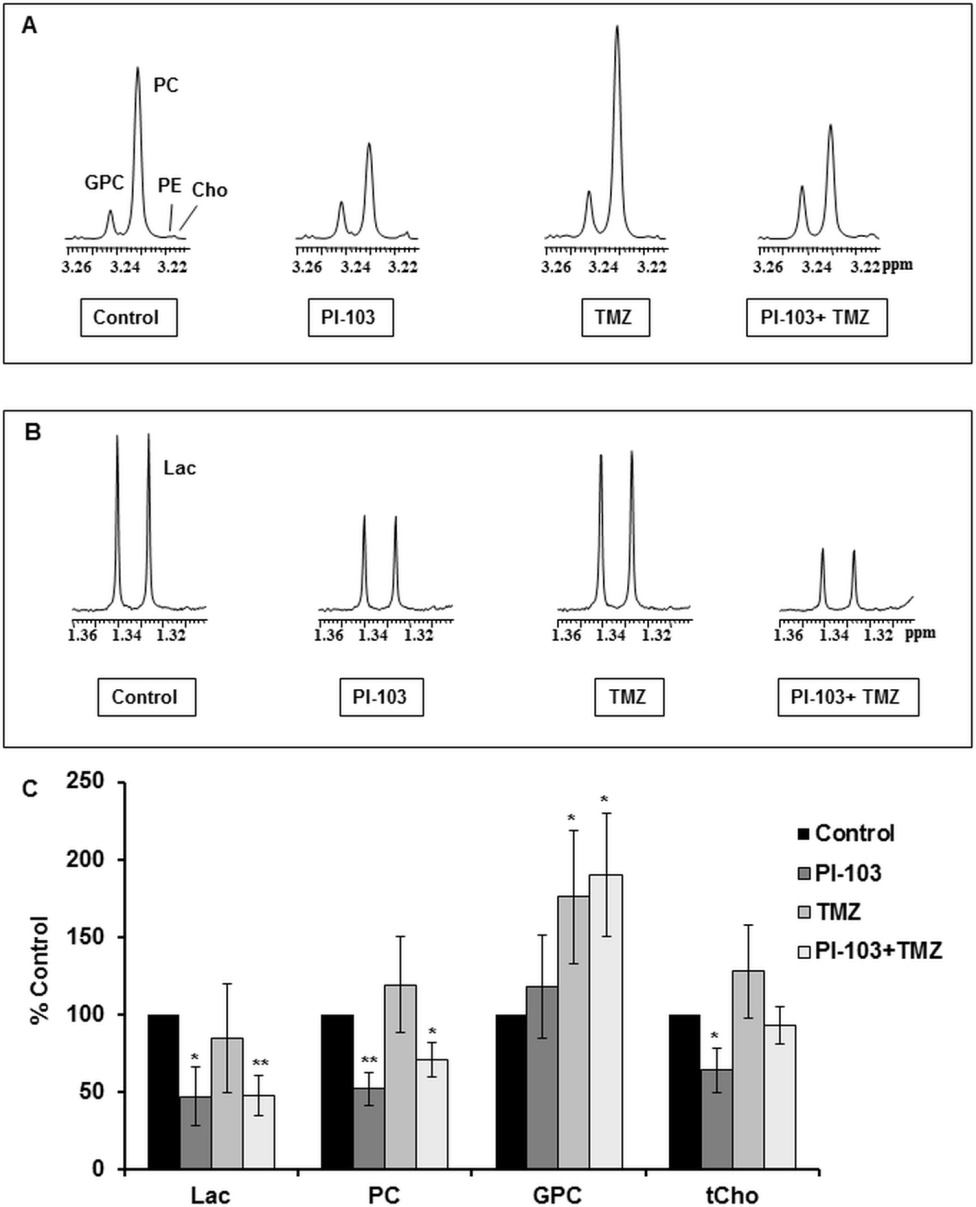
NMR-detected metabolic changes following treatment (48 h) with PI-103, TMZ or PI-103+TMZ in KNS42 paediatric glioblastoma cells. Representative ^1^H-NMR spectra of the aqueous cell extracts showing expanded regions for (A) Choline–containing metabolites or (B) Lactate. (C) A summary of ^1^H-NMR detected metabolic changes. Results are expressed as %Treatment/Control (T/C) and presented as the mean± SD (error bars) of at least three separate experiments. Statistically significantly different from the control *P≤0.05, **P≤0.01; two-tailed unpaired *t* test was used for all comparisons.

^1^H-NMR of aqueous extracts from control and treated cells was used to identify potential biomarkers for the combination of PI3K pathway inhibition with TMZ. Fig 2A and 2B show examples of the ^1^H-NMR spectra expansions of the tCho region consisting of PC+ GPC+ choline and the lactate peaks region and Table 2 summarizes the quantitative values of ^1^H-NMR detected metabolites of control, PI-103, TMZ or PI-103+ TMZ treated KNS42 cells. Analysis of ^1^H-NMR detected metabolic changes following single or combination drug treatments relative to vehicle treated controls (Fig 2C), showed that PI-103 treatment caused a decrease in lactate, PC and tCho but had no effect on GPC levels. By contrast, treatment with TMZ increased GPC levels with no effect on lactate, PC or tCho levels. Combination of PI-103 with TMZ resulted in a decrease in lactate and PC, and an increase in GPC levels. Opposite changes in PC and GPC resulted in no significant change in tCho in PI-103+ TMZ treated cells relative to control cells (*P* = 0.4). In addition, the PC/GPC ratio showed a significant decrease following treatment with PI-103 alone (2.4±0.7, *P* = 0.04) and in combination with TMZ (2.0±0.5, *P* = 0.03), but not with TMZ (3.7±1.1, *P* = 0.2), compared to control cells (5.4±1.7).

**Table 2:** Quantitative values of ^1^H-NMR-detected metabolites in control and treated (48 h) KNS42 paediatric glioblastoma cells.

Metabolites	lactate	PC	GPC	tCho
**DMSO**	5.5(±0.5)	30.2(±1.3)	7.0(±3.6)	38.1(±4.8)
**PI-103**	3.8(±0.6)*	18.7(±3.2)*	9.3(±4.7)	29.5(±6.9)
**TMZ**	3.4(±1.0)	24.9(±4.0)	8.4(±4.1)	34.1(±6.7)
**PI-103+ TMZ**	3.0(±0.9)*	21.1(±2.6)*	11.6(±3.4)	34.6(±2.7)

Data are expressed as mM and presented as the mean ± SD, n ≥ 3.

Two-tailed unpaired *t* test was used to compare results in treated cells to controls

*P≤0.05.

### Molecular effects of the combination of PI-103 with TMZ in SF188 paediatric glioblastoma cells

**Fig 3:**
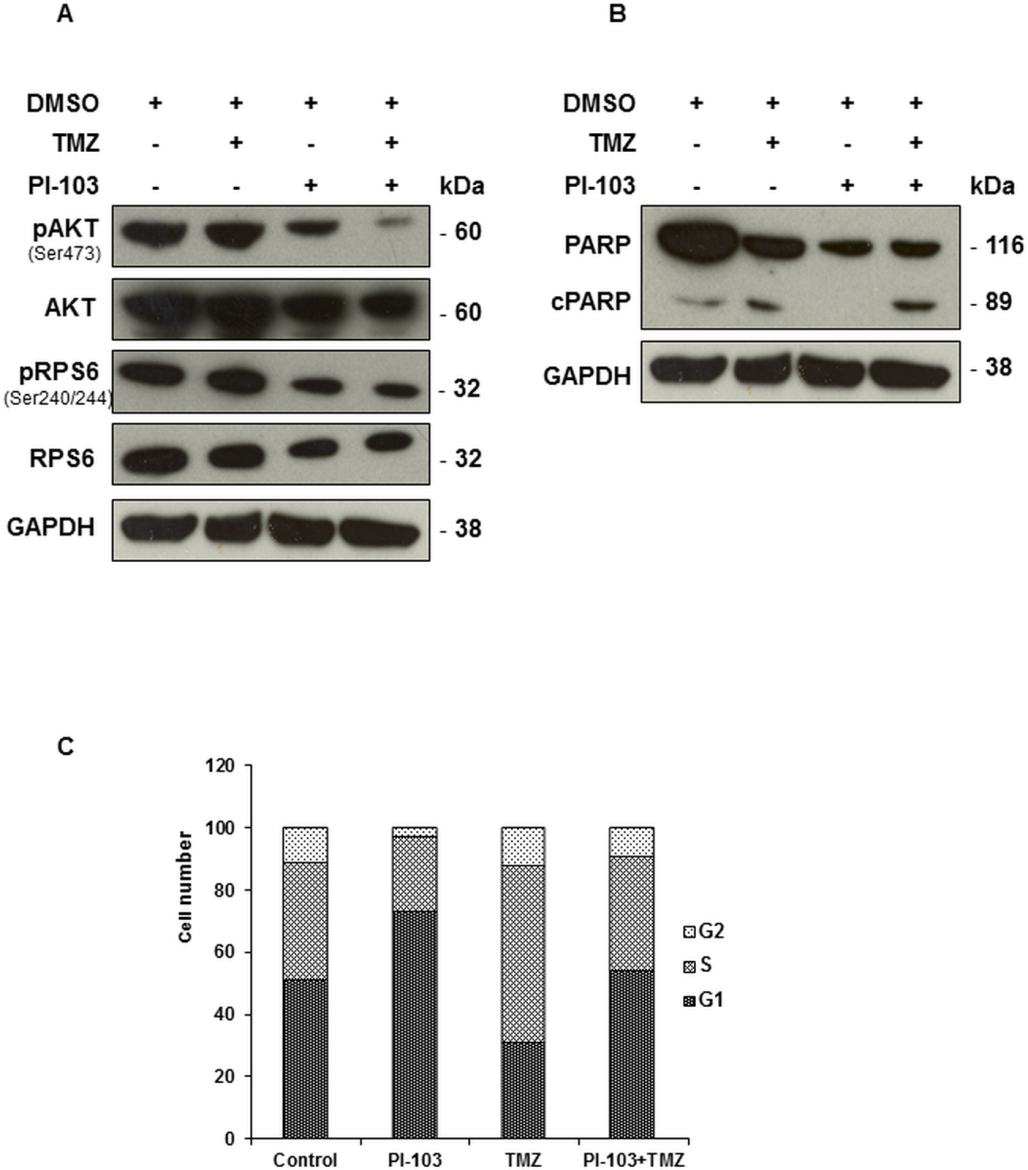
Molecular changes following treatment with (24 h) TMZ, PI-103 or PI-103+TMZ in SF188 paediatric glioblastoma cells. (A) Representative Western blots showing decreases in molecular biomarkers in the PI3K signalling pathway post-treatment relative to controls. (B) Representative Western blots showing induction of apoptosis as evidenced by the presence of cleaved PARP following treatment with TMZ or the combination of PI-103 with TMZ. GAPDH is used as a loading control. (C) A summary of the cell cycle distribution of control cells (DMSO), or following treatment.

To assess whether our NMR-detected biomarkers induced by treatment with PI-103 and TMZ in KNS42 cells could be detected in other paediatric glioblastoma cells with different genetic characteristics, we also investigated the effects of the combination of TMZ with PI-103 in SF188 human paediatric glioblastoma (WHO grade IV) cells. Published reports confirmed that SF188 cells are wildtype for *PTEN*, *PIK3CA* [12, 27] and histone H3.3 (H3F3A) G34V [30], and express MGMT [11, 12]. Immunoblotting analysis showed that treatment of SF188 cells with PI-103 for 24 h (2xGI_50_, GI_50_ = 0.2 μM) resulted in inhibition of the PI3K signalling pathway as shown by decreased phosphorylation of AKT (Ser473) and RPS6 (Ser240/244) in treated cells compared to their controls (Fig 3A, Table 3), while treatment with TMZ (2xGI_50_, GI_50_ = 460 μM) for 24 h induced apoptosis as shown by PARP cleavage (Fig 3B). Combination of PI-103 and TMZ resulted in inhibition of signalling downstream of PI3K and induced apoptosis (Fig 3A and 3B, Table 3). Densitometric analysis of protein bands showed a 2-fold and 5-fold increase cleaved PARP relative total PARP following TMZ or PI-103 +TMZ respectively, however both did not reach significance (P≥0.07; Table 3). Cell cycle analysis detected a G1 arrest (73±3% vs. 51±9%, *P* = 0.02) and a decrease cell population in the S phase (24±3% vs. 38±7%, *P* = 0.03) and G2 (3±1% vs. 11±2%, *P* = 0.002) phase following treatment with PI-103 relative to control cells (Fig 3C). However, treatment with TMZ resulted in an increase in the S phase (57±11% vs.38±7%, *P* = 0.05) and a decrease in the G1 phase (31±10% vs. 51±9%, *P* = 0.04; Fig 3C). As in the KNS42 cells, non-apoptotic cells remaining following treatment with the combination of both PI-103 and TMZ had a cell cycle profile similar to that of control cells (Fig 3C). We have previously reported that single agent treatment with PI-103 or TMZ reduced the number of treated cells per flask compared to controls (58±8%, *P* = 0.00007 and 66±17%, *P* = 0.008, respectively) [26]. Importantly, combination of PI-103 with TMZ caused a further reduction in cell number to 46±14%, *P* = 0.0004. Treatment with PI-103 and TMZ alone or in combination had no effect on the average cell volume (*P*≥0.09).

**Table 3:** Densitometric analysis of immunoblots from control and treated (24 h) SF188 paediatric glioblastoma cell extracts.

	pAKT(ser473)/AKT	pRPS6(Ser240/244)/RPS6	cPARP/PARP
**PI-103**	0.3(±0.3)*	0.3(±0.4)*	0.7(±0.5)
**TMZ**	1.2(±0.5)	1.0(±0.6)	2.4(±0.5)
**PI-103+ TMZ**	0.2(±0.04)*	0.4(±0.4)*	5.0(±2.3)

Data are expressed as fold changes relative to controls and presented as the mean ± SD, n ≥ 3.

Two-tailed unpaired *t* test was used to compare results in treated cells to controls

*P≤0.05.

### ^1^H-NMR detects similar metabolic changes following the combination of PI-103 with TMZ in SF188 compared to KNS42 paediatric glioblastoma cells

**Fig 4:**
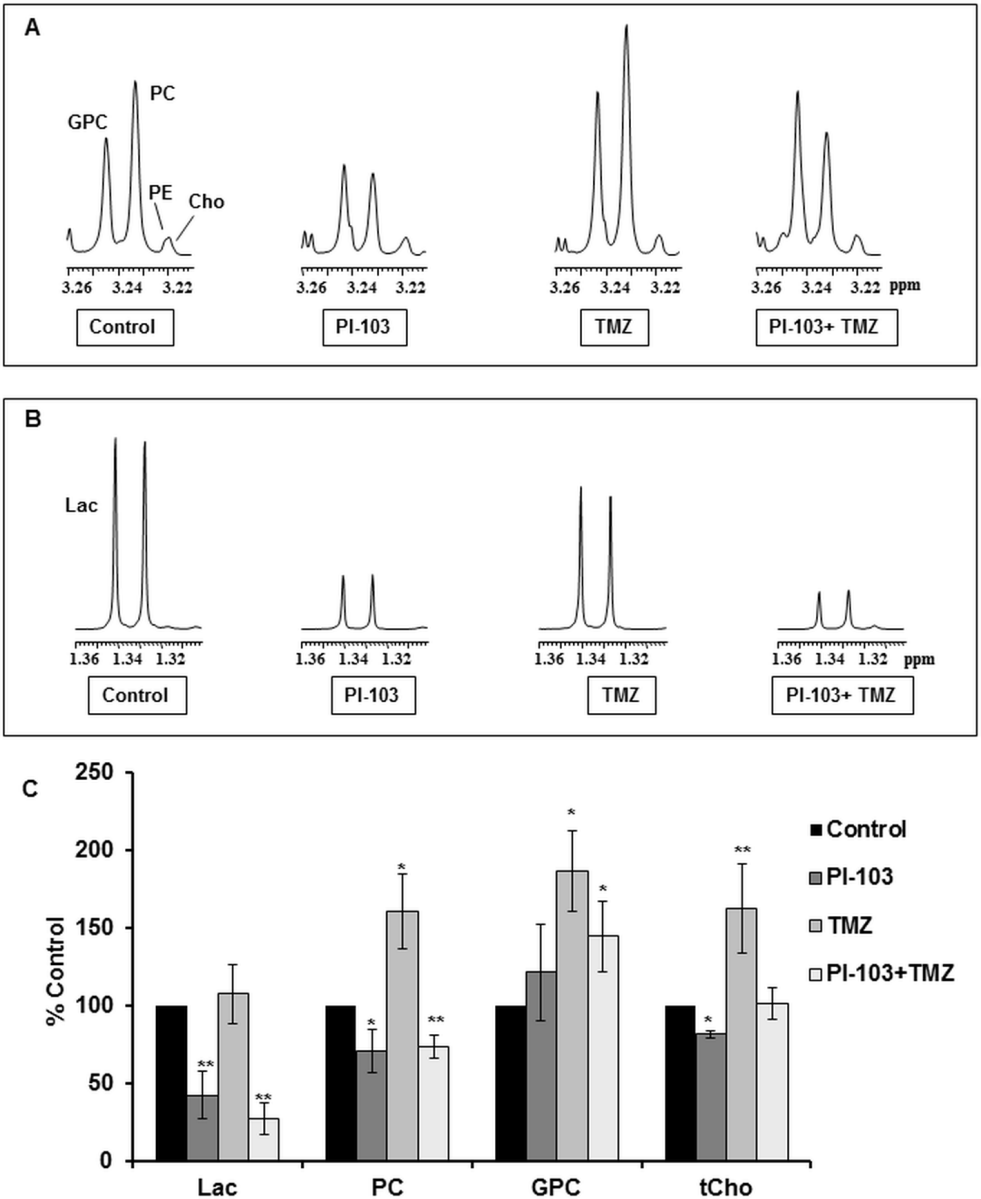
NMR-detected metabolic changes following treatment (24 h) with PI-103, TMZ or PI-103+TMZ in SF188 paediatric glioblastoma cells. Representative ^1^H-NMR spectra of the aqueous cell extracts showing expanded regions for (A) Choline–containing metabolites or (B) Lactate. (C) A summary of ^1^H-NMR detected metabolic changes. Results are expressed as %T/C and presented as the mean ± SD (error bars) of at least three separate experiments. Statistically significantly different from the control *P≤0.05, **P≤0.01; two-tailed unpaired *t* test was used for all comparisons.

Fig 4A and 4B show examples of the ^1^H-NMR spectra expansions of the tCho and lactate regions of control, PI-103, TMZ or PI-103+ TMZ treated SF188 cells and Table 4 summarizes the quantitative values of ^1^H-NMR detected metabolites. As we previously reported [26], analysis of the metabolic contents from the ^1^H-NMR spectra of PI-103 treated SF188 cells relative to their controls showed a decrease in lactate, PC and tCho levels (Fig 4C). Treatment with TMZ showed an increase in the levels of PC, GPC and tCho but lactate levels were not affected. In this study, combination of TMZ with PI-103 resulted in a decrease in lactate and PC levels but an increase in GPC, resulting in an overall level of tCho similar to that in control cells. Furthermore, a significant decrease in the PC/GPC ratio was detected following treatment with PI-103 alone (1.0±0.1, *P* = 0.01) and in combination with TMZ (0.9±0.04, *P* = 0.01), but not with TMZ (1.8±0.5, *P* = 0.8), compared to control cells (1.8±0.3).

**Table 4:** Quantitative values of ^1^H-NMR-detected metabolites in control and treated (24 h) SF188 paediatric glioblastoma cells.

Metabolites	lactate	PC	GPC	tCho
**DMSO**	42.5(±9.6)	9.6(±1.5)	5.5(±0.8)	16.2(±2.2)
**PI-103**	18.4(±4.4)*	7.1(±1.1)	6.9(±0.7)	15.9(±2.4)
**TMZ**	33.7(±7.2)	16.5(±8.3)	9.9(±5.9)	28.5(±15.0)
**PI-103+ TMZ**	11.7(±3.4)*	7.1(±0.9)	8.0(±0.7)*	16.7(±1.2)

Data are expressed as mM and presented as the mean ± SD, n ≥ 3.

Two-tailed unpaired *t* test was used to compare results in treated cells to controls

*P≤0.05.

### The combination of TMZ with PI-103 results in altered expression of enzymes involved in choline and glucose metabolism in both KNS42 and SF188 paediatric glioblastoma cells

**Fig 5:**
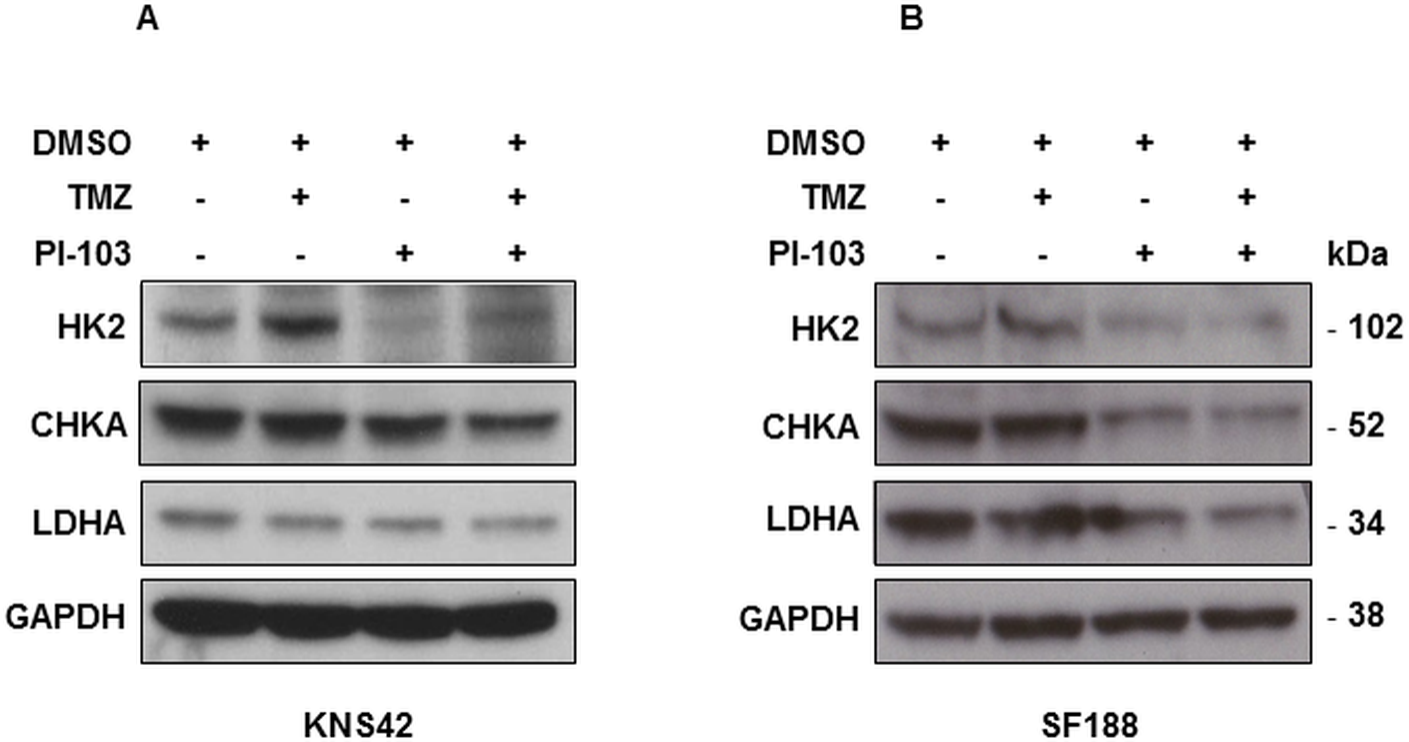
Changes in lactate and phosphocholine are associated with changes in protein levels of related enzymes. Representative Western blots showing changes in protein expression levels of enzymes involved in choline metabolism (CHKA) and glucose metabolism (HK2 and LDHA) post-treatment relative to controls in: (A) KNS42 or (B) SF188. GAPDH is used as a loading control.

Previously we have shown that our NMR-detected metabolic changes were associated with alterations in enzymes involved in choline and glucose metabolism following PI3K/mTOR signalling pathway inhibition [26]. We have used immunoblotting to identify the effects of combining PI3K/mTOR inhibition with TMZ on these enzymes. Both in KNS42 and SF188 cells, a decrease in CHKA expression levels compared to control cells was observed following treatment with PI-103 and the combination but not with TMZ alone (Fig 5A and 5B, Table 5). Furthermore, a reduction in the protein expression levels of the glycolytic enzyme HK2 was detected following treatment with PI-103 and the combination (Fig 5A and 5B, Table 5). The glycolytic enzymes LDHA also showed a decrease following treatment with PI-103 but did not reach significance in KNS42 cells (*P* = 0.07); however combination of PI-103 with TMZ resulted in a significant decrease in LDHA (*P* = 0.04) in both cell lines (Fig 5A and 5B, Table 5).

**Table 5:** Densitometric analysis of immunoblots from control and treated paediatric glioblastoma cell extracts.

	KNS42			SF188		
	HK2	LDHA	CHKA	HK2	LDHA	CHKA
**PI-103**	0.4(±0.2)*	0.7(±0.2)	0.5(±0.1)*	0.6(±0.2)*	0.8(±0.1)*	0.8(±0.1)*
**TMZ**	1.2(±0.3)	0.8(±0.2)	0.9(±0.6)	1.2(±0.3)	1.1(±0.1)	0.9(±0.2)
**PI-103+ TMZ**	0.5(±0.3)*	0.8(±0.1)*	0.3(±0.2)*	0.7(±0.1) *	0.6(±0.2)*	0.6(±0.1) *

Data are expressed as fold changes relative to controls and presented as the mean ± SD, n ≥ 3.

Two-tailed unpaired *t* test was used to compare results in treated cells to controls

*P≤0.05.

## Discussion

It is generally well established that combinations of anti-cancer treatments offering complementary mechanisms and synergistic pharmacodynamic interactions can be more effective in anti-cancer therapy than single-target approaches [31, 32]. The PI3K-AKT-mTOR pathway is activated in both adult and paediatric glioblastoma, and contributes to resistance to the standard-of-care chemotherapeutic agent TMZ [12, 33, 34]. Increasing evidence suggested clinical benefits are obtained by combining TMZ with inhibitors of the PI3K-AKT-mTOR pathway for the treatment of adult and paediatric glioblastoma ([12, 35] and references therein). Identification of non-invasive biomarkers of target inhibition and potentially of tumour response to this novel combination treatment would be of value in the clinical development of combination treatments, in particular for the treatment of childhood brain tumours, where repeated biopsy post-administration of a new treatment is typically too invasive and therefore not routinely carried out. We investigated whether a combination of the dual pan-Class I PI3K/mTOR inhibitor PI-103 [13–15] with TMZ in paediatric glioblastoma cell lines would show changes in their metabolic profiles that could be detected with NMR. Furthermore, we aimed to identify mechanisms underlying the detected metabolic changes.

Using two genetically distinct paediatric glioblastoma cell lines, KNS42 and SF188, inhibition of the PI3K signalling pathway with PI-103 resulted in a cell cycle arrest in G1 phase and a decrease in cellular growth, and was associated with a significant decrease in lactate, PC and tCho levels detected by ^1^H-NMR. This is consistent with previously published results by us and others [26, 36–40], using different PI3K/AKT/mTOR inhibitors in various cancer types including adult and paediatric glioblastoma. Treatment of KNS42 or SF188 cells with the DNA damaging agent TMZ induced an S phase cell cycle arrest and apoptosis. Furthermore, it induced a distinct metabolic effect compared to treatment with PI-103 including an increase in GPC levels. An increase in GPC levels has been previously reported following treatment with other anti-cancer drugs [36, 40, 41].

As the two compounds have different mechanisms of action, adding the effects of both monotherapies together is expected to yield a contribution from each compound. Simultaneous treatment of cells with PI-103 and TMZ, at a concentration equivalent to 2xGI_50_ of each drug, resulted in a further decrease (11–25%) in cell number compared to individual treatments in both models of paediatric glioblastoma. This could be attributed to the induction of apoptosis. However, immunoblotting did not show increased apoptosis, as indicated by cleaved PARP, following the combination compared to single TMZ treatment. This might be due to the fact that we have only included the attached cells in our immunoblotting analysis, and therefore we have not accounted for the late apoptotic cells which could be present in the growth medium of cells treated with PI-103 and TMZ.

The cellular effects were reflected in the metabolic changes. Co-treatment with PI-103 and TMZ resulted in a combination of the biomarker changes induced by each individual treatment, including a decrease in lactate, PC and an increase in GPC levels in both KNS42 and SF188 cells. Combining the two agents resulted in no significant change in the tCho peak compared to controls due to the opposing metabolic changes including the decrease in PC and increase in GPC levels, caused by PI-103 and TMZ respectively. On the other hand, a decrease in PC/GPC ratio was detected in both paediatric glioblastoma KNS42 and SF188 cells following treatment with PI-103 alone and in combination with TMZ. Since a decreased PC/GPC ratio is associated with a lower malignancy [42], our results demonstrate that treatment with PI-103 alone or in combination with TMZ alters the choline metabolite profile of paediatric glioblastoma cells towards a less malignant phenotype.

We have also investigated the mechanisms underlying our NMR-detected metabolic changes. This may provide a means of understanding the downstream metabolic consequences on cell signalling pathways and may also help identify potential targets for new agents. The PI3K/AKT/mTOR signalling pathway is a master regulator of enzymes involved in glucose, glutamine and lipid metabolism [43, 44]. Therefore, inhibition of the PI3K signalling pathway is expected to impact on levels/activities of these enzymes. In line with this, we have shown that the decrease in PC following treatment with PI-103 alone or in combination with TMZ was associated with a reduction in the protein expression level of CHKA, the enzyme responsible for choline phosphorylation to form PC. Furthermore, a decrease in the protein expression levels of the glycolytic enzymes HK2 and LDHA was also detected following PI-103 treatment alone and with the combination, suggesting that PI-103 interferes with glycolysis causing a decrease in the production of lactate.

Regarding the clinical translation potential of the metabolic biomarkers detected in our system, the use of MRS to non-invasively monitor metabolic changes in response to therapy in glioma is well established both in adult and paediatric patients [21, 22, 45–47]. This involves monitoring changes in PC and GPC levels with ^31^P-MRS or using ^1^H-MRS to detect lactate and tCho. Innovative ^31^P-MRS approaches based on ^1^H/ ^31^P polarization transfer are being developed to improve *in vivo* detection and quantification of phosphomonoesters including phosphoethanolamine (PE) and PC, and phosphodiesters including glycerophosphoethanolamine (GPE) and GPC, non-invasively on small animal and clinical MR scanners [45, 48, 49]. Concerning the detection of lactate, many mechanisms could contribute to the steady-state lactate signal that is observed using ^1^H-MRS *in vivo*, and hence interpreting lactate using this methodology remains complex [50]. Improved techniques using multiple quantum filters have been demonstrated in clinical studies and provide more robust measurements [51]. Hyperpolarized ^13^C-MRS methodologies have emerged as another approach, with promising results, for *in vivo* monitoring of metabolism of different types of cancer including glioblastoma [52–54]. A recent study showed a significantly lower hyperpolarized lactate-to-pyruvate ratio in animals treated with the PI3K/mTOR inhibitor voxtalisib, TMZ, or combination therapy, which when compared with controls was associated with enhanced animal survival [55].

Because early paediatric clinical trials of new PI3K or PI3K/mTOR inhibitors, as single agents and in combination with other cancer treatments, are planned in glioblastoma, we have focussed on this tumour type in our current work and we are building on this by investigating inhibition *in vivo* in orthotopic models of paediatric glioblastoma established in our Unit. This strategy provides a closer model to the application of these non-invasive techniques in the paediatric brain. *In vivo* investigation also requires a PI3K pathway inhibitor suitable for clinical studies that will also cross the blood brain barrier. PI-103 does not fulfil these criteria. Recent studies using adult glioblastoma models demonstrated that combinatorial treatment with TMZ and the PI3K/mTOR inhibitors voxtalisib or NVP-BEZ235 significantly reduced tumour growth rates and prolonged median survival of tumour-bearing mice [35, 55]. Our *in vivo* studies combining PI3K pathway inhibition with cytotoxic cancer treatments in subcutaneous and intracranial paediatric tumours continue and will constitute a publication in their own right.

In conclusion, we have shown that the *in vitro* combination of PI3K/mTOR inhibition and TMZ in paediatric glioblastoma cells, independent of molecular characteristics, caused alteration in lactate and choline metabolite levels detected by NMR. Although the metabolites identified in this study cannot be conclusively defined as biomarkers for monitoring response to this combination therapy, they do have the potential to become biomarkers once these results have been confirmed *in vivo*, ideally using orthotopic tumour models.
